# Crohn’s and Colitis Canada’s 2021 Impact of COVID-19 and Inflammatory Bowel Disease in Canada: Epidemiology—The Trends of Disease Over Time

**DOI:** 10.1093/jcag/gwab029

**Published:** 2021-11-05

**Authors:** Stephanie Coward, Joseph W Windsor, M Ellen Kuenzig, Alain Bitton, Charles N Bernstein, Jennifer L Jones, Reena Khanna, Kate Lee, Sanjay K Murthy, Laura Targownik, Eric Benchimol, James Guoxian Huang, Mariam S Mukhtar, Parul Tandon, Gilaad G Kaplan

**Affiliations:** 1 Department of Medicine, University of Calgary, Calgary, Alberta, Canada; 2 Department of Community Health Sciences, University of Calgary, Calgary, Alberta, Canada; 3 SickKids Inflammatory Bowel Disease Centre, Division of Gastroenterology, Hepatology and Nutrition, The Hospital for Sick Children, Toronto, Ontario, Canada; 4 Child Health Evaluative Sciences, SickKids Research Institute, Toronto, Ontario, Canada; 5 Department of Medicine, McGill University Health Centre, McGill University, Canada; 6 Department of Internal Medicine, Max Rady College of Medicine, Rady Faculty of Health Sciences, University of Manitoba, Winnipeg, Manitoba, Canada; 7 University of Manitoba IBD Clinical and Research Centre, Winnipeg, Manitoba, Canada; 8 Department of Medicine, Dalhousie University, Halifax, Nova Scotia, Canada; 9 London Health Sciences Centre–University Campus, Western University, London, Ontario, Canada; 10 Crohn’s and Colitis Canada, Toronto, Ontario, Canada; 11 The Ottawa Hospital IBD Centre, Department of Medicine, University of Ottawa, Ottawa, Ontario, Canada; 12 Division of Gastroenterology and Hepatology, Mount Sinai Hospital, University of Toronto, Toronto, Ontario, Canada; 13 ICES, Toronto, Ontario, Canada; 14 Department of Paediatrics and Institute of Health Policy, Management and Evaluation, University of Toronto, Toronto, Ontario, Canada; 15 Department of Internal Medicine, King Abdulaziz University Hospital, Jeddah, Saudi Arabia

**Keywords:** Crohn’s disease, coronavirus, epidemiology, inflammatory bowel disease, reproduction number, ulcerative colitis

## Abstract

At the beginning of the coronavirus disease 2019 (COVID-19) pandemic, there were many unknowns: transmission vectors of the virus, appropriate intervention strategies and if being immunocompromised due to inflammatory bowel disease (IBD), for example, or medications put a person at increased risk for severe COVID-19. Imposing and relaxing of public health restrictions at different times and in different regions in Canada led to different epidemiologies of the virus in different provinces and territories. In order to understand the waxing and waning of waves of the COVID-19 pandemic, it is necessary to understand the effective reproductive number (*R*_*t*_) and the countervailing forces that exert upward or downward pressure on the spread of the virus at a given point in time. As many regions in Canada deal with a third wave, the primary forces affecting the *R*_*t*_ of severe acute respiratory syndrome coronavirus 2 are variants of concern and the increasing vaccinations of Canadians leading to increased population-level immunity. Fortunately, for the IBD population, current research suggests that those with IBD are not at increased risk of contracting COVID-19, nor of having a more severe disease course when compared to the general population.

**Key Messages**

- The transmissibility (*R*_*t*_) of SARS-CoV-2 is affected by the basic properties of the virus, modified by public health measures and uptake of vaccination, which can be measured over time.
- There is no significant increase in risk for contracting COVID-19 or having severe COVID-19 for people with IBD compared to the general population.
- The major risk factors for severe COVID-19 among people with IBD continue to be advanced age or severe disease activity requiring high-dose (>20 mg/day) corticosteroid usage.

## Introduction

In December of 2019, China’s Hubei province diagnosed dozens of pneumonias from an unknown cause (1). These cases of severe acute respiratory syndrome (SARS) were identified as a novel coronavirus (CoV) named SARS-CoV-2. By January 20, 2020, SARS-CoV-2 was recognized outside of China in Thailand, Japan and South Korea (2). As of January 23, 2020, a large-scale public health lockdown of Wuhan, a city of 11 million people, was instituted to quarantine an epidemic. On February 11, 2020, the World Health Organization (WHO) named the illness caused by infection with SARS-CoV-2 ‘corona virus disease 2019 (COVID-19)’ (1); then, on March 11, 2020, the WHO declared COVID-19 a global pandemic (3). A week later, China reported no new domestic cases for first time since the outbreak began—the end of China’s first wave marked the beginning of the first wave in countries throughout the world (1). This article explains the impact of the reproductive number of SARS-CoV-2 on the epidemiology of COVID-19, describes the Canadian experience with COVID-19 during the first year of the pandemic and explores the epidemiology of COVID-19 in those with inflammatory bowel disease (IBD).

## THE EPIDEMIOLOGIC DETERMINATES OF A PANDEMIC: REPRODUCTIVE NUMBER

The rate and extent of the spread of SARS-CoV-2 across different geographic populations is a property of the virus’ reproductive number. The basic reproductive number (*R*_0_) reflects the transmissibility of a virus (i.e., the average number of secondary infections caused by the primary case) in the absence of interventions to control the virus when the whole population is susceptible to infection (4). When the *R*_0_ exceeds 1, infections increase throughout the population. Without any interventions to control the spread of SARS-CoV-2, its *R*_0_ is 5.7 (possibly higher for variants of concern), more than five times that of seasonal influenza (~1.3); this highlights the highly infective nature of SARS-CoV-2 that allowed the virus to cause a global pandemic (5). Features of SARS-CoV-2 that make the virus highly infectious include mode of transmission (droplets and airborne), incubation period (~5 days) prior to symptom onset, asymptomatic or mild symptoms in the majority of those infected and the novelty of SARS-CoV-2 making the entire population susceptible. Additionally, the recent emergence of more infectious variants of concern has further increased the transmissibility of SARS-CoV-2 (2).

SARS-CoV-2 causes severe disease in a subset of individuals, particularly in those of advanced age or with multiple comorbidities. At the beginning of the pandemic, the case-fatality (i.e., the number of individuals who died relative to the number diagnosed with COVID-19) was 2% to 3%, which was more than 20-fold higher than that of seasonal influenza (0.1%) (6). During the first wave of the pandemic, the net effect of the high morbidity and mortality associated with COVID-19 led to overwhelmed health care systems in the United States (e.g., New York City) and Italy (e.g., Milan) (7). By April 2020, daily deaths from COVID-19 in the United States surpassed any other cause, including cancer, cardiovascular disease and motor vehicle accidents. Moreover, models predicted catastrophic numbers of cases, hospitalizations, ICU admissions and deaths if the virus were allowed to run rampant throughout society without any intervention (8).

**Figure 1.**
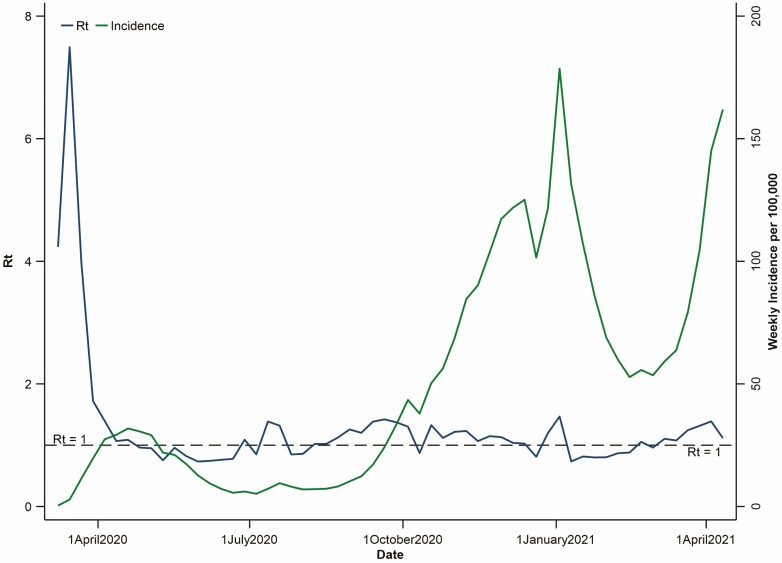
*R*_*t*_ of COVID-19 in Canada (blue) and weekly incidence of new cases per 100,000 total population (green). *R*_*t*_ is the effective reproduction number (https://royalsociety.org/-/media/policy/projects/set-c/set-covid-19-R-estimates.pdf) and represents the rate of transmission of the virus at a given point in time (4). *R*_*t*_ was calculated based on a weekly time interval, considering the doubling time and exponential growth of COVID-19 in Canada. This figure demonstrates how minor fluctuations in *R*_*t*_ can lead to exponential growth (or decline) in weekly incidence of cases and gives credence to the need to drive *R*_*t*_ below 1 to manage the pandemic. As seen in the most recent data, with *R*_*t*_ decreasing but still above 1, the rate of new cases can be slowed, but *R*_*t*_ must fall below 1 before the number of weekly cases begins to decline. Weekly case counts for both incidence and *R*_*t*_ calculated from Government of Canada, April 20, 2021 (https://www.canada.ca/en/public-health/services/diseases/2019-novel-coronavirus-infection.html).

Due to the morbidity and mortality of SARS-CoV-2, governments across the world instituted public health measures to drive down the effective reproduction number (*R*_*t*_). *R*_*t*_ is a measure of the actual transmissibility of a virus at a given point in time when measures are taken to slow an infection rate as opposed to the basic *R*_0_, which is a measure of how infectious a virus is in the absence of contravention. In order to bring the *R*_*t*_ below 1, at which point infections in the population decrease, public health agencies recommended drastic interventions that governments instituted, including stopping travel between countries; hygiene activities (such as handwashing); physical distancing beyond 2 m; personal protective equipment for health care professionals; wearing masks by the general population; work from home for non-essential occupations; closure of schools and transition to online learning; limits to in-person gatherings; and the closure of non-essential businesses such as entertainment, fitness and restaurants. At the peak of the first wave of the pandemic, the majority of Canada’s population lived under these conditions, and adherence to public health recommendations on limiting interpersonal contact and avoiding non-essential movement was high. These early efforts were successful in reducing the *R*_*t*_ below 1, leading to the end of the first wave by the summer of 2020, to where the number of cases and deaths occurring daily in Canada were measured in the hundreds and tens, respectively (Figure 1).

As governments relaxed restrictions at the trough of each wave of COVID-19, though, increased contact between individuals allowed SARS-CoV-2 to re-emerge within society (an increase in *R*_*t*_) leading to a second wave of infections, which became evident in the early fall of 2020 (Figure 1). Rising infections in parallel to easing restrictions is a product of low infection rates in the general population as models projected that over two-thirds of Canadians required natural immunity (i.e., herd immunity) to keep the *R*_*t*_ of SARS-CoV-2 below 1 without public health intervention. By the end of 2020, two opposing factors would pull against the *R*_*t*_ of SARS-CoV-2: vaccines and variants (9).

The spring of 2021 ushered the second wave of the pandemic in Canada. Driving new cases are variants of concerns—mutations of SARS-CoV-2 that increase the transmissibility of the virus. These variants are easier to acquire and, in turn, raise the *R*_*t*_ of SARS-CoV-2. Counterbalancing the pressure of new cases is (i) the escalating vaccination of Canadians from those on the frontlines to those who are most vulnerable to the general population and (ii) ongoing public health measures that continue to protect the public from infections. The tug-of-war between variants and vaccines, as well as the actions of the public to prevent transmission, will define the height of the subsequent waves of the COVID-19 pandemic in Canada and ultimately the waning of SARS-CoV-2 from the world.

## EPIDEMIOLOGY OF COVID-19 IN CANADA

The global epidemiology of COVID-19 is comprehensively maintained by the Center for Systems and Engineering at Johns Hopkins University’s interactive online dashboard (https://www.arcgis.com/apps/dashboards/index.html#/bda7594740fd40299423467b48e9ecf6) (10). On March 19, 2020, over 230,000 individuals were diagnosed with COVID-19 with 9300 deaths reported. As of April 29, 2021, there have been more than 130 million global cases and over 2.8 million deaths. While SARS-CoV-2 reached virtually every populated region on Earth, the impact of COVID-19 varied by geography.

**Figure 2.**
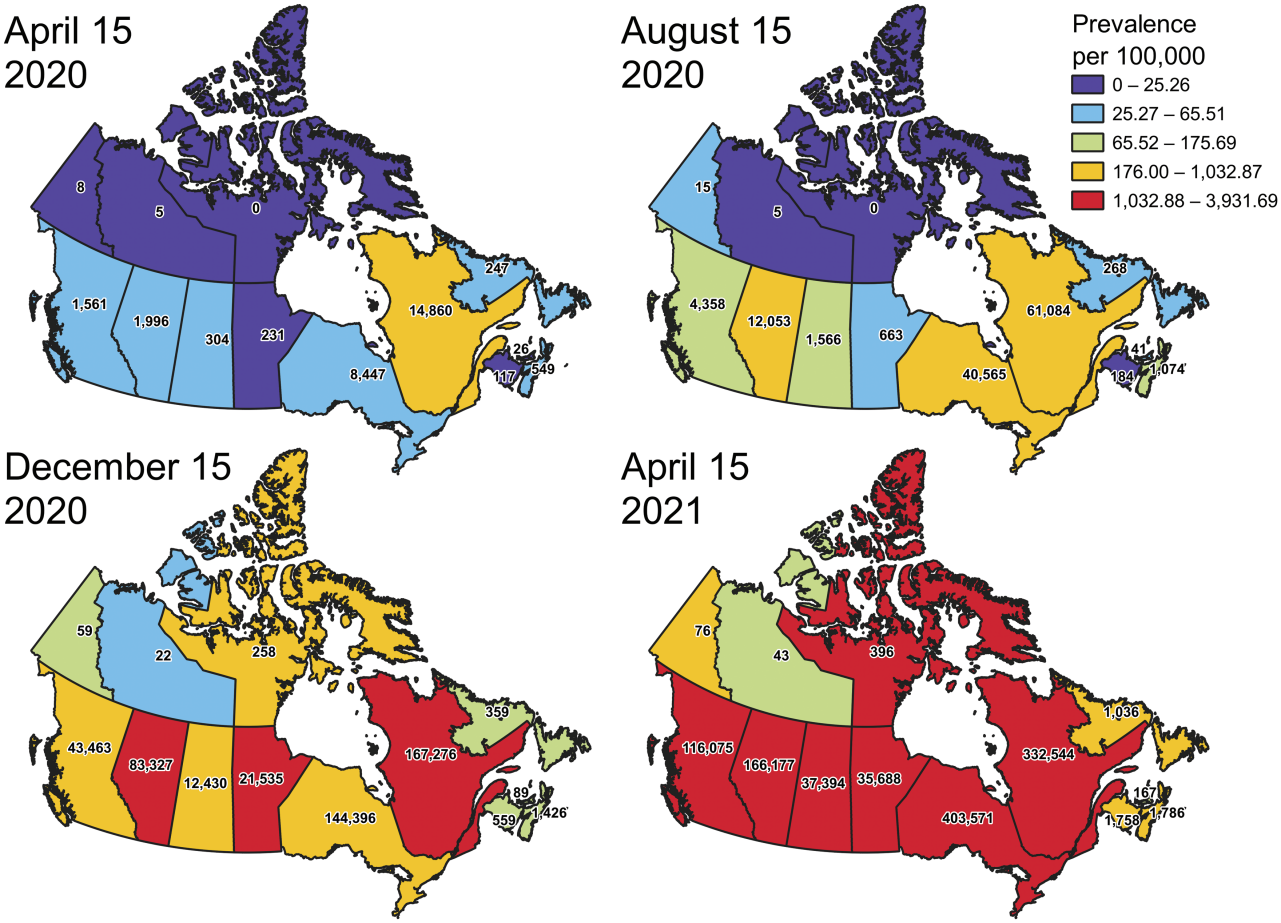
Prevalence of COVID-19 cases in Canada per 100,000 population over time. Total cumulative COVID-19 cases (numbers on each province/territory) and prevalence per 100,000 population (2019 population) colour-coded by quintiles from April 15, 2020–April 15, 2021. The prevalence of COVID-19 in Canada changed in different regions throughout the pandemic due to adherence to public health measures: For example, while Nova Scotia was originally one of the hardest-hit provinces by the virus, the *R*_*t*_ of the virus was dramatically reduced after the first wave of the pandemic such that the Atlantic Bubble (Nova Scotia, New Brunswick, Prince Edward Island and Newfoundland and Labrador) did not experience a dramatic second wave, worse than the first, at the same time as most central and western provinces experienced their second waves. Daily cumulative totals retrieved from Government of Canada, April 19, 2021 (https://www.canada.ca/en/public-health/services/diseases/2019-novel-coronavirus-infection.html).

On January 25, 2020, the first case of COVID-19 was reported in Canada, an individual who travelled in Wuhan, China. During the first 2 months of 2020, COVID-19 cases in Canada were driven largely by those returning from travel outside of the country. However, by March 2020, community spread was recorded in most provinces. The COVID-19 Canadian Outbreak Tracker provided by Esri Canada is an online interactive dashboard (https://resources-covid19canada.hub.arcgis.com/) that reported the number of diagnosed Canadians with COVID-19 throughout the pandemic. On March 19, 2020, the database reported 846 Canadians diagnosed with COVID-19, which escalated to over 1 million cases and 23,000 deaths in the span of just over one year—by April 3, 2021. The per capita rates of COVID-19 in the first wave of the pandemic were highest in Quebec and Ontario, followed by the West and Nova Scotia; the rest of the Atlantic provinces, the prairie provinces and the territories showed the lowest infection rates per capita during the first wave of the pandemic (Figure 2).

In response to the threat SARS-CoV-2 overwhelming the ability of the Canadian health care systems to provide life-preserving care for the infected, every provincial government instituted policies under the guidance of their medical officers of health to reduce the opportunities for viral transmission; this included the closing of non-essential business, closing in-person instruction at schools and universities and shifting to remote learning, and imposing restrictions on mobility and social contact between households. The sacrifices made by Canadians to lockdown during the first wave of the pandemic were effective in flattening the epidemiologic curve of COVID-19 and preventing overwhelmed health care systems whereby admissions to hospital and to intensive care units would have otherwise surged past capacity. The pooled effect of these restrictions, as well as the high adherence of the population to these restrictions, reduced transmissibility of SARS-CoV-2, allowing time for health care systems to increase capacity, procure additional personal protective equipment, implement testing and contact tracing protocols, and refine our understanding of how SARS-CoV-2 is spread and the impact of mask wearing on reducing transmission. During this time, we also continued to gather knowledge on how to best improve outcomes for persons with COVID-19, and to begin the trials that would eventually lead to the development of highly efficacious vaccines.

Throughout Canada, the second wave became apparent in October 2020. To our worst fears, the number of cases in the fall was considerably higher than the first wave in the spring: During the first wave of the pandemic (March to May 2020), daily cases peaked at ~1500 diagnoses a day, whereas over 8000 cases a day were reported across Canada in early January 2021. The second wave led to reimplementation of lockdowns that ultimately reduced the number of cases of COVID-19 in February 2021. As restrictions following the second wave were lifted, we have entered a third wave with case counts exceeding those at the peak of the second wave with new cases reaching more than 9000 per day in April 2021.

**Figure 3.**
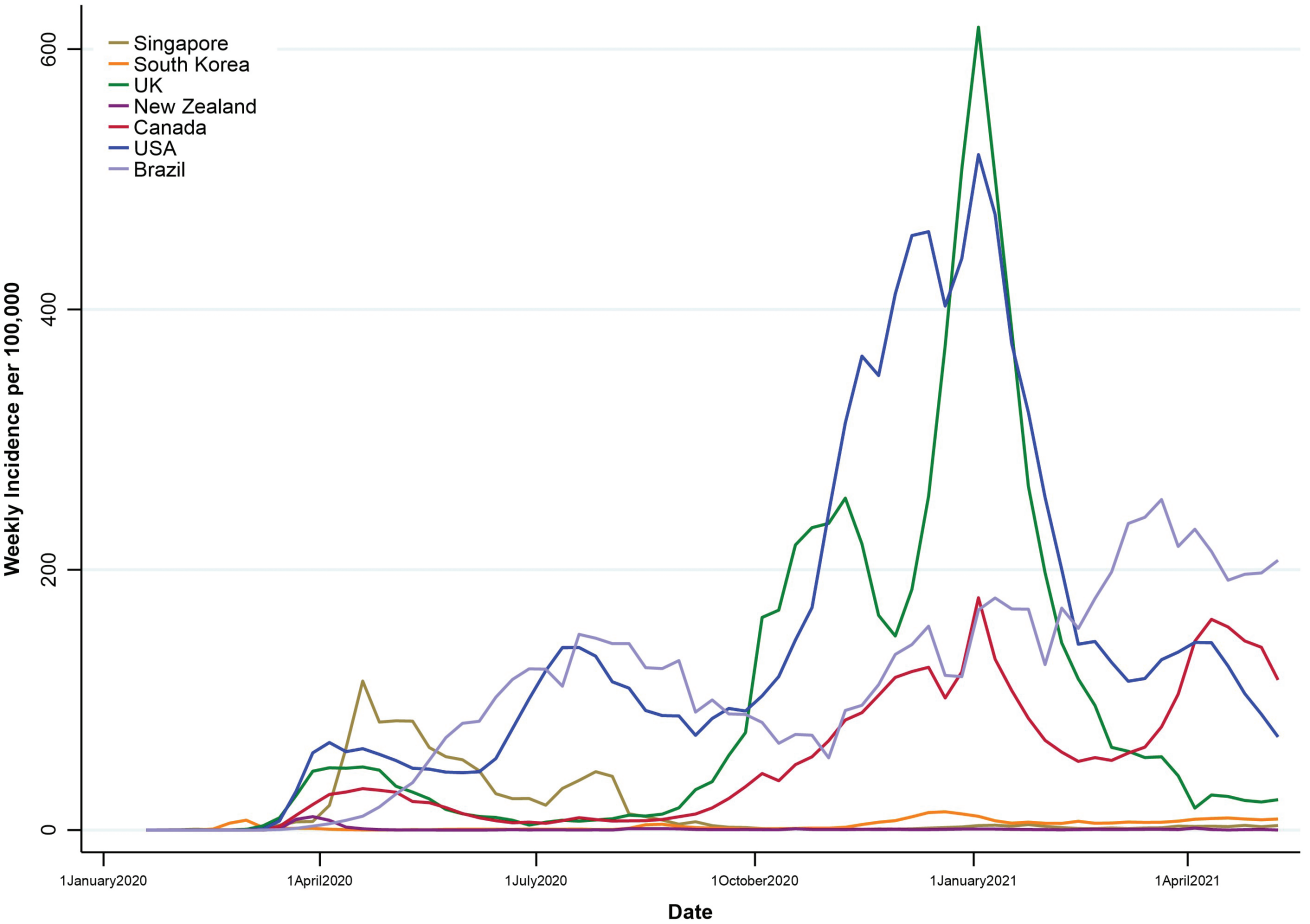
Weekly incident cases in select countries representative of various regions around the world. Weekly incidence per country calculated using data from Johns Hopkins University’s COVID-19 Dashboard by the Center for Systems Science and Engineering (May 18, 2021) and estimated 2020 regional populations from worldometers.info/world-population/population-by-country/ (April 9, 2021). Many nations around the world quickly reacted to the SARS-CoV-2 outbreak by instituting public health measures in the face of paucity of information about the virus; while so-called lockdown countries like New Zealand, Singapore and South Korea faired quite well with the virus after the first wave, other countries like Brazil, the United Kingdom and the United States saw exponential growth in viral incidence. Other important correlates that can be seen in this graphs are the rise in cases following premature easing of restrictions, such as the April 2020 easement of restrictions in Brazil; the effectiveness of strict lockdowns in response to outbreaks such as in the case of Singapore’s swift response to incidence peaks in Spring 2020; dramatic increase in weekly cases following community transmission of variants of concern, such the September 2020 identification of the B.1.1.7 variant in the United Kingdom; and the effectiveness of vaccines (first vaccination in the United Kingdom: December 8, 2020; first vaccinations in Canada and the United States: December 14, 2020) and national masking initiatives (April 14, 2020 in Singapore, July 10–14, 2020 in various regions of the United Kingdom, and January 20, 2021 in the United States).

Relative to many other nations, Canada has been somewhat mediocre in mitigating the spread of SARS-CoV-2: We have not enjoyed the success of countries that swiftly implemented strict lockdown laws like New Zealand, Singapore or South Korea; but neither have we experienced the overwhelming spikes witnessed in countries that prematurely eased restrictions like Brazil, the United Kingdom or the United States. The efforts of Canadians to isolate and reduce the spread allowed us to avoid overwhelming our health care systems during the first wave of the pandemic (March to June 2020). However, at the time of writing (May 18, 2021), we are currently in the midst of our third wave, which had higher daily case counts than the peak of our second wave in January 2021. If anything can be learned from the global epidemiology of this virus, it is that as vaccine roll-out continues, we will be able to drive weekly case counts down sharply as we get nearer to herd immunity. Figure 3 provides a comparison of selected countries with Canada, including some key milestones that influence *R*_*t*_ in those regions.

## EPIDEMIOLOGY OF COVID-19 IN PEOPLE WITH IBD

When the first wave of the pandemic hit Canada in the spring of 2020, individuals with IBD were alarmed by the unknown risk of COVID-19 among the immunocompromised. In the absence of any data on the risk of COVID-19 in persons with IBD, many gastrointestinal societies provided recommendations to persons with IBD that aligned with public health recommendations to reduce transmission (11–14). Early analyses in Asia and Europe suggested that these public health measures may have reduced transmission of SARS-CoV-2 in the IBD population (15,16).

Most studies suggest that the risk of acquiring COVID-19 is the same across the IBD and general populations. A systematic review of 24 studies showed a lower incidence of COVID-19 infection in those with IBD (4.02 per 1000) as compared to the general population (6.59 per 1000); however, this difference in risk was not statistically significant (17). A nationwide study in Denmark during the first wave of the pandemic showed those with IBD were less susceptible to being diagnosed with COVID-19 as compared to the general population (18), whereas a population-based study from Sweden showed that persons with IBD were more likely to be hospitalized for COVID-19, but the risk of severe disease (ICU admission or death) was similar to the general population (19). Similarly, the risk of hospitalization or death after COVID-19 in those with IBD was similar to the general population in a U.S. population (20). An Italian study reported that people with IBD were more likely to become infected with COVID-19 and more likely to require hospitalization than the general population (21). A key limitation of these studies is their failure to account for potential differences in adherence to public health guidelines (e.g., working from home) among the IBD population relative to the general population and if reassuring data on risk from the first wave of the pandemic influenced these behaviours during the second wave.

A systematic review of on the prevalence of COVID-19 in those with autoimmune diseases demonstrated that those with IBD had the lowest prevalence among immune-mediated diseases (22). Serosurveillance studies in IBD populations without a known diagnosis of COVID-19 have shown a seroprevalence of 1% in Canada (23), which was lower than that in the United States (3%) (24) and the United Kingdom (4.3%) (25). Overall, the prevalence of COVID-19 in those with IBD likely corresponds to the frequency of SARS-CoV-2 in the general population; for example, in Milan (an epicentre for COVID-19), 5.4% of people with IBD had antibodies to SARS-CoV-2 as compared to only 0.4% in regions in Italy with lower rates of infection in their general populations (26). Serosurveillance studies in those with IBD should be interpreted cautiously as the CLARITY study in the United Kingdom demonstrated that seroconversion was influenced by therapy whereby those on anti-TNF (3.4%) had a lower seropositivity as compared to those on vedolizumab (6.0%) (25). Additionally, a study from Canada showed serological response wanes over time in persons with IBD who recovered from an infection from SARS-CoV-2 (23).

## CONCLUSION

The epidemiology of SARS-CoV-2 and the disease caused by a resulting infection, COVID-19, is complicated by the waxing and waning of public health measures instituted in different regions at different times, and local compliance with those measures. Different regions around the world serve as case studies for the effectiveness of various public health measures such as masking, hand hygiene and lockdowns. With the exception of localized outbreaks that overwhelmed some health systems early in the pandemic, general adherence to public health guidelines have bought health researchers the necessary time to discover treatments and vaccines to SARS-CoV-2 and to understand the definition of vulnerable populations to this viral threat: In the early stages of the pandemic, it was not clear if those with IBD would be at greater risk of infection or severe disease; over the course of the past year, we have learned that those with IBD at the greatest risk of severe COVID-19 are those of advanced age and those on high-dose steroids for their IBD. As we proceed through the second year of this pandemic, the most pressing questions for the IBD community concern vaccines, the amount of immune response mounted by people on immunomodulating therapies for their IBD and how long immunity can be conferred by prior infection or vaccination in this population.

## References

[1] Wu Z , McGoogan JM. Characteristics of and important lessons from the coronavirus disease 2019 (COVID-19) outbreak in China: Summary of a report of 72 314 cases from the Chinese Center for Disease Control and Prevention. J Am Med Assoc 2020;323(13):1239–42. 10.1001/jama.2020.2648 32091533

[2] Wiersinga WJ , Rhodes A, Cheng AC, et al. Pathophysiology, transmission, diagnosis, and treatment of coronavirus disease 2019 (COVID-19): A review. J Am Med Assoc 2020;324(8):782–93. 10.1001/jama.2020.12839 32648899

[3] WHO Director-General. WHO Director-General’s opening remarks at the media briefing on COVID-19—11 March 2020. 2020. <https://www.who.int/dg/speeches/detail/who-director-general-s-opening-remarks-at-the-media-briefing-on-covid-19---11-march-2020> (Accessed May 5, 2021).

[4] The Royal Society. Reproduction number (R) and growth rate (r) of the COVID-19 epidemic in the UK: Methods of estimation, data sources, causes of heterogeneity, and use as a guide in policy formulation. 2020. <https://royalsociety.org/-/media/policy/projects/set-c/set-covid-19-R-estimates.pdf> (Accessed May 5, 2021).

[5] Sanche S , Lin YT, Xu C, et al. High contagiousness and rapid spread of severe acute respiratory syndrome coronavirus 2. Emerg Infect Dis 2020;26(7):1470–7. 32255761 10.3201/eid2607.200282 PMC7323562

[6] Piroth L , Cottenet J, Mariet AS, et al. Comparison of the characteristics, morbidity, and mortality of COVID-19 and seasonal influenza: A nationwide, population-based retrospective cohort study. Lancet Respir Med 2021;9(3):251–9. 33341155 10.1016/S2213-2600(20)30527-0 PMC7832247

[7] Oster AM , Kang GJ, Cha AE, et al. Trends in number and distribution of COVID-19 hotspot counties—United States, March 8–July 15, 2020. Morb Mortal Wkly Rep 2020;69(33):1127–32. 10.15585/mmwr.mm6933e2 PMC7439980 32817606

[8] Weitz JS , Beckett SJ, Coenen AR, et al. Modeling shield immunity to reduce COVID-19 epidemic spread. Nat Med 2020;26(6):849–54. 32382154 10.1038/s41591-020-0895-3 PMC8272982

[9] Kissler SM , Tedijanto C, Goldstein E, et al. Projecting the transmission dynamics of SARS-CoV-2 through the postpandemic period. Science 2020;368(6493):860–8. 32291278 10.1126/science.abb5793 PMC7164482

[10] Dong E , Du H, Gardner L. An interactive web-based dashboard to track COVID-19 in real time. Lancet Infect Dis 2020;20(5):533–4. 32087114 10.1016/S1473-3099(20)30120-1 PMC7159018

[11] Rubin DT , Abreu MT, Rai V, et al.; International Organization for the Study of Inflammatory Bowel Disease. Management of patients with Crohn’s disease and ulcerative colitis during the coronavirus disease-2019 pandemic: Results of an international meeting. Gastroenterology 2020;159(1):6–13.e6. 32272113 10.1053/j.gastro.2020.04.002 PMC7194599

[12] British Society of Gastroenterology. BSG multi-society guidance on further recovery of endoscopy services during the post-pandemic phase of COVID-19. 2021. <https://www.bsg.org.uk/covid-19-advice/bsg-multi-society-guidance-on-further-recovery-of-endoscopy-services-during-the-post-pandemic-phase-of-covid-19> (Accessed May 5, 2021).

[13] Crohn’s and Colitis Foundation. COVID-19 (coronavirus): What IBD patients should know. 2021. <https://www.crohnscolitisfoundation.org/coronavirus/what-ibd-patients-should-know> (Accessed May 5, 2021).

[14] Crohn’s and Colitis Canada. COVID-19 and IBD recommendations. 2021. <https://crohnsandcolitis.ca/Living-with-Crohn-s-Colitis/COVID-19-and-IBD> (Accessed May 5, 2021).

[15] An P , Ji M, Ren H, et al. Prevention of COVID-19 in patients with inflammatory bowel disease in Wuhan, China. Lancet Gastroenterol Hepatol 2020;5(6):525–7. 10.1016/S2468-1253(20)30121-7 PMC7164865 32311321

[16] Fiorino G , Gilardi D, Radice S, et al. Absence of COVID-19 infection in patients accessing IBD unit at Humanitas, Milan: Implications for postlockdown measures. Am J Gastroenterol 2020;115(10):1719–21. 32852334 10.14309/ajg.0000000000000829 PMC7499879

[17] Singh AK , Jena A, Kumar-M P, et al. Risk and outcomes of coronavirus disease in patients with inflammatory bowel disease: A systematic review and meta-analysis. United European Gastroenterol J 2021;9(2):159–76. 10.1177/2050640620972602 PMC8250629 33210980

[18] Attauabi M , Poulsen A, Theede K, et al. Prevalence and outcomes of COVID-19 among patients with inflammatory bowel disease—a Danish prospective population-based cohort study. J Crohns Colitis 2021;15(4):540–50. 33035299 10.1093/ecco-jcc/jjaa205 PMC7797764

[19] Ludvigsson JF , Axelrad J, Halfvarson J, et al. Inflammatory bowel disease and risk of severe COVID-19: A nationwide population-based cohort study in Sweden. United European Gastroenterol J 2021;9(2):177–92. 10.1002/ueg2.12049 PMC8014882 33704918

[20] Singh S , Khan A, Chowdhry M, et al. Risk of severe coronavirus disease 2019 in patients with inflammatory bowel disease in the United States: A multicenter research network study. Gastroenterology 2020;159(4):1575–8.e4. 32522507 10.1053/j.gastro.2020.06.003 PMC7702184

[21] Carparelli S , Pastore MR, Valvano MR, et al. Worse impact of second wave COVID-19 pandemic in adults but not in children with inflammatory bowel disease: An Italian single tertiary center experience. Eur Rev Med Pharmacol Sci 2021;25(6):2744–7. 33829460 10.26355/eurrev_202103_25437

[22] Akiyama S , Hamdeh S, Micic D, Sakuraba A. Prevalence and clinical outcomes of COVID-19 in patients with autoimmune diseases: A systematic review and meta-analysis. Ann Rheum Dis 2020. Online ahead of print. doi:10.1136/annrheumdis-2020-218946. 33051220

[23] Kaplan GG , Ma C, Charlton C, Khanj J, Tipples G, Panaccione R. Antibody response to SARS-CoV-2 among individuals with inflammatory bowel disease diminishes over time: A serosurveillance cohort study. Gut 2021:gutjnl-2021-325238. PMID:34340997. Online ahead of print. doi:10.1136/gutjnl-2021-325238 PMC9120372 34340997

[24] Gubatan J , Levitte S, Balabanis T, et al. SARS-CoV-2 testing, prevalence, and predictors of COVID-19 in patients with inflammatory bowel disease in Northern California. Gastroenterology 2020;159(3):1141–4.e2. 32387541 10.1053/j.gastro.2020.05.009 PMC7204754

[25] Kennedy NA , Goodhand JR, Bewshea C, et al.; Contributors to the CLARITY IBD Study. Anti-SARS-CoV-2 antibody responses are attenuated in patients with IBD treated with infliximab. Gut 2021;70(5):865–75. 33753421 10.1136/gutjnl-2021-324388

[26] Berte’ R , Mazza S, Stefanucci MR, et al. Seroprevalence of SARS-CoV-2 in IBD patients treated with biologic therapy. J Crohns Colitis 2021;15(5):864–8. 33211810 10.1093/ecco-jcc/jjaa237 PMC7717179

